# Machine Learning Approach to Reduce Alert Fatigue Using a Disease Medication–Related Clinical Decision Support System: Model Development and Validation

**DOI:** 10.2196/19489

**Published:** 2020-11-19

**Authors:** Tahmina Nasrin Poly, Md.Mohaimenul Islam, Muhammad Solihuddin Muhtar, Hsuan-Chia Yang, Phung Anh (Alex) Nguyen, Yu-Chuan (Jack) Li

**Affiliations:** 1 Graduate Institute of Biomedical Informatics College of Medical Science and Technology Taipei Medical University Taipei Taiwan; 2 International Center for Health Information Technology Taipei Medical University Taipei Taiwan; 3 Research Center of Big Data and Meta-analysis Wan Fang Hospital Taipei Medical University Taipei Taiwan; 4 Department of Healthcare Information & Management Ming Chuan University Taoyuan City Taiwan; 5 Department of Dermatology Wan Fang Hospital Taipei Taiwan; 6 TMU Research Center of Cancer Translational Medicine Taipei Medical University Taipei Taiwan

**Keywords:** clinical decision support system, alert fatigue, machine learning, artificial neural network

## Abstract

**Background:**

Computerized physician order entry (CPOE) systems are incorporated into clinical decision support systems (CDSSs) to reduce medication errors and improve patient safety. Automatic alerts generated from CDSSs can directly assist physicians in making useful clinical decisions and can help shape prescribing behavior. Multiple studies reported that approximately 90%-96% of alerts are overridden by physicians, which raises questions about the effectiveness of CDSSs. There is intense interest in developing sophisticated methods to combat alert fatigue, but there is no consensus on the optimal approaches so far.

**Objective:**

Our objective was to develop machine learning prediction models to predict physicians’ responses in order to reduce alert fatigue from disease medication–related CDSSs.

**Methods:**

We collected data from a disease medication–related CDSS from a university teaching hospital in Taiwan. We considered prescriptions that triggered alerts in the CDSS between August 2018 and May 2019. Machine learning models, such as artificial neural network (ANN), random forest (RF), naïve Bayes (NB), gradient boosting (GB), and support vector machine (SVM), were used to develop prediction models. The data were randomly split into training (80%) and testing (20%) datasets.

**Results:**

A total of 6453 prescriptions were used in our model. The ANN machine learning prediction model demonstrated excellent discrimination (area under the receiver operating characteristic curve [AUROC] 0.94; accuracy 0.85), whereas the RF, NB, GB, and SVM models had AUROCs of 0.93, 0.91, 0.91, and 0.80, respectively. The sensitivity and specificity of the ANN model were 0.87 and 0.83, respectively.

**Conclusions:**

In this study, ANN showed substantially better performance in predicting individual physician responses to an alert from a disease medication–related CDSS, as compared to the other models. To our knowledge, this is the first study to use machine learning models to predict physician responses to alerts; furthermore, it can help to develop sophisticated CDSSs in real-world clinical settings.

## Introduction

Initiation of computerized provider order entry (CPOE) systems has allowed physicians to order medications, laboratory tests, and other ancillary services electronically [1]. CPOE systems create an opportunity to improve patient care by decreasing medication errors, reducing redundant test orders, and promoting standardized clinical practice [2,3]. However, CPOE is often integrated with a clinical decision support system (CDSS) in order to make better clinical decisions through guidance, alerts, and reminders. A CDSS is always combined with software algorithms that generate alerts during orders entered into a CPOE by physicians [4,5]. Each of these alerts addresses a meaningful clinical issue relevant to the administration process and has a positive impact on identifying and preventing erroneous or less optimal prescription [6-8].

The productivity of CDSSs is often impaired by generating distracting alerts in the system (ie, a high volume of clinically irrelevant alerts) [9,10]. van der Sijs et al [11] suggested that an ideal CDSS should have high specificity and sensitivity, provide clear information, and facilitate safe and efficient handling of alerts. A recent study reported that approximately 90%-95% of medication alerts are overridden by providers [12,13], and more than half of overrides are due to alerts being deemed clinically irrelevant [14]. The main concern is that these large numbers of clinically irrelevant alerts might cause alert fatigue and consume too much time and mental energy. Moreover, it sometimes leads staff to override both critical warnings and unimportant alerts. Getting frequent false alerts can desensitize physicians so that providers always ignore and mistrust alerts with acceleration [15]. Ignoring clinically relevant alerts too much triggers patient harm and is associated with an increased rate of mortality.

Until now, significant efforts and strategies have been implemented in minimizing alert fatigue, such as the administration of highly specific algorithms [16], customization of third-party providers’ sets of alerts [17], and execution of tiered severity grading to stratify and lessen the number of false alerts [18]. Several studies suggested turning off frequently overridden alerts [19], updating clinical content to deliver the most current evidence at the point of care, and holding consensus meetings between physicians and pharmacists [20]. Since physicians increasingly adopt electronic prescribing, the progression and proclamation of CDSS alerts might depend, in part, on whether providers find medication safety alerts valuable.

Machine learning is comprised of a collection of techniques that has the potential to learn complex rules and to identify patterns from multidimensional datasets. It has been effectively employed in many areas, such as disease risk prediction [21], classification [22], and health care utilization [23]. To our knowledge, no studies have examined machine learning techniques regarding medication alert reduction in a large number of alert analyses among physicians of different specialties. We hypothesized that machine learning models could predict physician responses, which would ultimately directly assist in developing a sophisticated CDSS for reducing alert fatigue. Therefore, the primary objective of this study was to develop and validate machine learning models to reduce alert fatigue by predicting physician responses. This study may provide perspective on the perceived usefulness of CDSS alerts in patient care and insights into how to design better alert systems in real-world clinical settings. It can contribute to minimizing the number of alerts in the user interface, ensuring the appropriate prescription, and reducing the severity of unintended consequences.

## Methods

### Ethical Approval and Study Process

**Figure 1:**
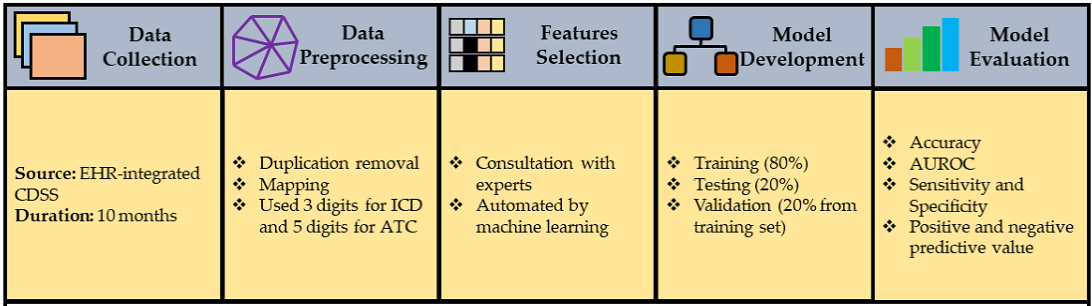
Study design process. ATC: Anatomical Therapeutic Chemical classification system; AUROC: area under the receiver operating characteristic curve; CDSS: clinical decision support system; EHR: electronic health record; ICD: International Classification of Diseases.

This type of study does not require Institutional Review Board review, following the policy of the National Health Research Institutes in Taiwan, as it provides a large amount of computerized, deidentified data. The entire study process is shown in Figure 1.

### Data Source

We collected data from an electronic health record (EHR)–integrated disease medication–related CDSS from a university teaching hospital in Taiwan. We considered only prescriptions that generated alerts due to a prescription error in the CDSS. The data collection period was between August 2018 and May 2019. During the 10-month study period, 9213 prescriptions generated alerts that accounted for approximately 3% of total prescriptions provided by physicians.

### Data Preprocessing

The first step of this study was to clean the data. In the dataset, lots of duplications of prescriptions appeared, which means there were several prescriptions with the same patient’s registration number, diagnosis code (ie, International Classification of Diseases, 10th Revision, Clinical Modification [ICD-10-CM]), and drug code (ie, Anatomical Therapeutic Chemical [ATC] classification system code). Therefore, we removed those prescriptions and kept the most recent prescription. A total of 6453 prescriptions were considered to develop machine learning–based prediction models. A prescription with the Taiwan National Health Insurance code as the diagnosis code was mapped to the ICD-10-CM code. Data normalization was carried out by converting all the values between 0 and 1. Finally, the data were converted into a matrix that included the diagnosis code, drug code, department ID, and physician ID.

### Feature Selection

There could be more than 20 different clinical variables available in a single prescription. Therefore, feature selection is essential in order to keep the variables within a manageable size to be able to optimize the prediction model. The feature selection process was completed in three stages: (1) consultation with an expert (YL) who is a physician and specialist in CDSSs, (2) automated feature selection via machine learning algorithms, and (3) reduction of the number of input variables by using only the first three digits of the diagnosis code (ie, ICD-10-CM) and the first five digits of the drug code (ie, ATC). The patient’s age, the patient’s gender, the diagnosis code (ie, ICD-10-CM), the drug code (ie, ATC), the physician ID, and the department ID were considered as input variables. We then created a matrix for the diagnosis code (ie, ICD-10-CM), the drug code (ie, ATC code), the physician ID, and the department ID. A total of 6453 input variables were used to develop a machine learning model with binary outcomes.

**Table 1:** List of input variables.

Variable	Input column contents	Input column number
Patient’s gender	Male or female	1
Diagnosis code	First 3 digits of the ICD-10-CM^a^ code	822
Drug code	First 5 digits of the ATC^b^ classification system code	262
Physician ID	Physician ID	227
Department ID	Department ID	29

^a^ICD-10-CM International Classification of Diseases, 10th Revision, Clinical Modification.

^b^ATC: Anatomical Therapeutic Chemical.

### Model Development

#### Overview

**Figure 2:**
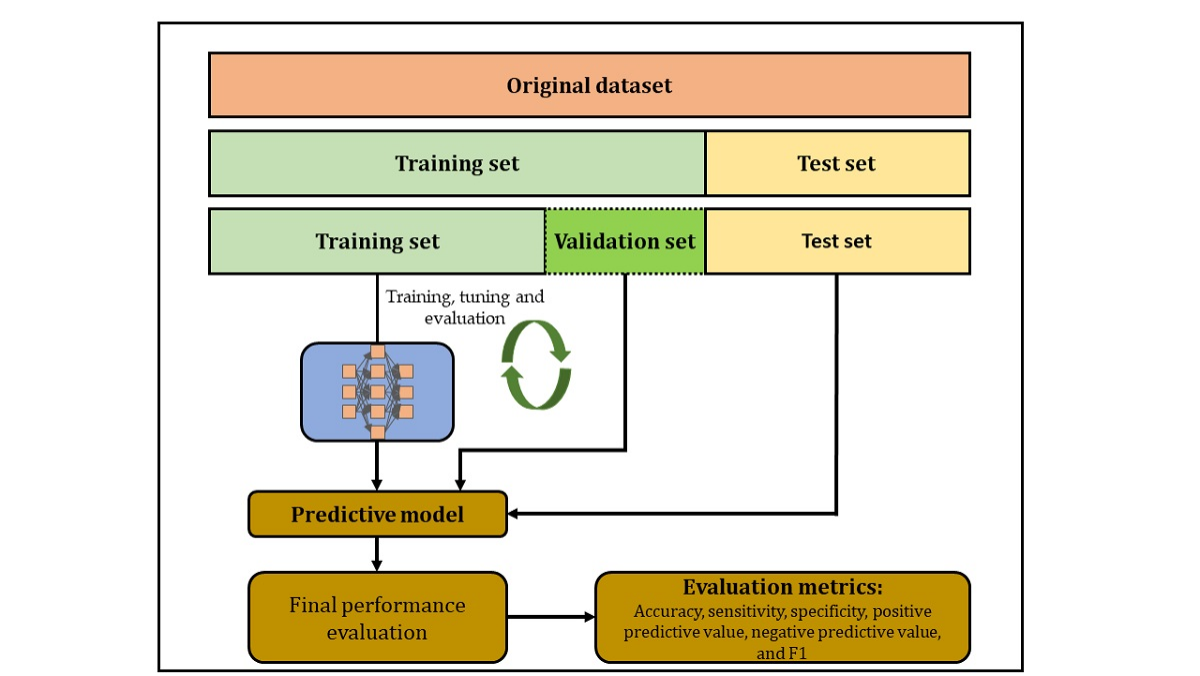
Distribution of training and testing datasets for model development.

The objective of the model was to reliably predict what would be physicians’ responses to an alert. We divided the entire dataset into two parts: (1) the training dataset (80% of the dataset) and (2) the testing dataset (20% of the dataset). However, the model was trained using 60% of the dataset as the training set and 20% of the dataset as an internal validation set. The remaining 20% of the dataset was used for testing our model’s performance (see Figure 2). Model development was carried out using Python 3.6 software (Python Software Foundation). Python is a free and open-source programming language and environment for statistical computing and graphics.

#### Artificial Neural Network

Artificial neural networks (ANNs) were first introduced in the 1940s; recently, they have become extremely powerful and one of the most popular machine learning models that interconnects with adaptive simple processing elements. They usually work by mimicking the biological nervous systems responsible for knowledge processing and knowledge representation [24]. ANN-based algorithms have already shown high performance in terms of accuracy, sensitivity, and specificity for classification problems. Therefore, the application of ANNs has increased globally in recent years in health care research, including in drug development, pattern recognition, disease prediction, disease diagnosis, and disease prognosis. ANNs consist of three layers of neurons: the *input layer,* the *hidden layers,* and the *output layer*. The hidden layer can be a single or multiple layer. Every hidden layer is comprised of an activation function. In our study, we used three hidden layers, with the *rectified linear unit* (ReLU) activation function in the first and second hidden layers, and the *sigmoid* activation function in the third hidden layer.

The ReLU is a widely used activation function in the prediction model. It converts input values from 0 to α. In the third layer, we used a sigmoid activation function due to a nonlinear nature. The sigmoid function is also one of the most commonly used activation functions for binary classification. The sigmoid function converts output classes between 0 and 1. The ANN was designed to be a classification model that can predict the responses from multiple physicians while minimizing prediction errors by using *binary cross-entropy* as loss function and the stochastic gradient descent method for optimization. Moreover, 100 epochs were used in the ANN model where maximum accuracy and minimum loss for training and validation can be achieved.

#### Random Forest

Random forest (RF) is also known as *ensemble learning* because it is an ensemble of a large number of individual decision trees [25]. Each tree in the RF model spits out a class prediction, and the class with the most votes becomes our model’s prediction. However, RF applies to both the *classification* and *regression* models.

#### Naïve Bayes

**Figure 3:**
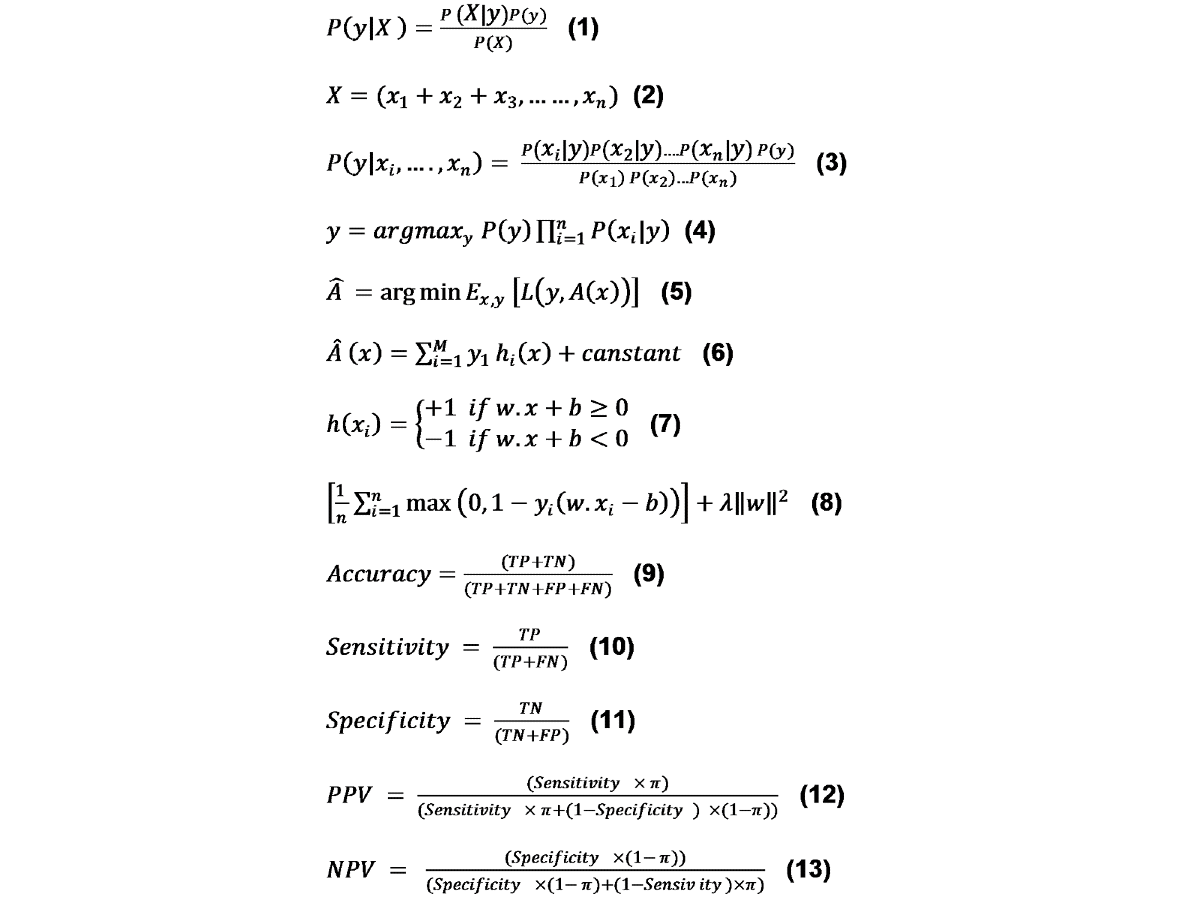
Equations. FN: false negative; FP: false positive; NPV: negative predictive value; PPV: positive predictive value; TN: true negative; TP: true positive.

Naïve Bayes (NB) is a classification model that uses the *Bayesian probability* theory during prediction [26]. It is also known as a probabilistic classifier. In 1960, the NB model was first introduced for text classification by the text retriever community [27]. However, there are several types of NB algorithms for parameter estimation and event models, such as *Gaussian naïve Bayes*, *multinomial naïve Bayes*, and *Bernoulli naïve Bayes*. Bayes theorem is expressed as equation 1 in Figure 3.

The variable *y* is the class variable that represents whether the alert will be accepted or rejected given the condition. Variable *X* represents the features like drugs, disease, and demographic. *X* is given as equation 2 in Figure 3.

Here, *x*_1_, *x*_2_ ... *x*_n_ represent the features (ie, they can be mapped to outcome: accept or reject alert). By substituting for *X* and expanding using the chain, the rule is given in equation 3 in Figure 3. In our model, the class variable *y* has two outcomes: accept or reject. There could be cases where the classification is multivariable. Therefore, the equation 4 in Figure 3 is used to find the class variable *y* with maximum probability.

#### Gradient Boosting

Gradient boosting (GB) is one of the promising machine learning algorithms that has already shown better prediction for classification [28]. It can be used both in classification and regression models. Like RF, GB is a set of decision trees, but the main differences are how the trees are built and how the results are combined. In the RF model, each tree is built independently, while in the GB model they are built one tree at a time. The GB model works in a forward stage-wise manner and converts weak learners to strong learners [29]. The most interesting part of the GB algorithm is that it can easily fit into the new model. Moreover, the RF model combines results at the end of the process, by averaging or *majority rules*, while the GB model combines results along the way [30].

In the training set, input variables such as drugs and diseases, make a set {(*x*_1_*y*_1_), ... ,(*x_n_y_n_*)} of known values of *x* and corresponding values of *y*. The goal is always to find an approximation *Â* (*x*) to find a function *A* (*x*) that minimizes the expected value of the specified loss function *L*(*y*, *A*(*x*)), as shown in equation 5 in Figure 3.

The GB model assumes a real-valued *y* and calculates an approximation *Â* (*x*) in the form of a weighted sum of functions *h_i_*(*x*) from *H* classes, which are called base or weak learners, as shown in equation 6 in Figure 3.

#### Support Vector Machine

Support vector machine (SVM) is a supervised machine learning algorithm. SVM is used both in *classification* and *regression* problems [31]. It is also used to solve linear and nonlinear problems and works well for many complex problems. The idea of SVM is simple: it creates a line or a hyperplane that separates the data into classes. The hypothesis function *h* is defined as shown in equation 7 in Figure 3.

The point above or on the hyperplane is classified as a class +1, and the point below the hyperplane is classified as a class –1. The SVM classifier works in the form shown in equation 8 in Figure 3.

### Model Performances

#### Overview

To evaluate the performance of five machine learning algorithms, we calculated accuracy, sensitivity, specificity, positive predictive value (PPV), negative predictive value (NPV), and the area under the receiver operating characteristic curve (AUROC). For calculating those measures, we measured true positive, true negative, false positive, and false negative. The definitions of the six parameters are given below.

#### Accuracy

Accuracy is the test by which we can see how accurate our model is. The equation to calculate accuracy is shown in equation 9 in Figure 3.

#### Sensitivity

Sensitivity is the test by which we can determine a positively identified case. The equation to calculate sensitivity is shown in equation 10 in Figure 3.

#### Specificity

Specificity is the measure by which we can measure correctly identified cases from negative cases. The mathematical equation is given in equation 10 in Figure 3.

#### PPV and NPV

PPV and NPV are two basic measures in biomedical studies. PPV is the probability that the positively identified case is positive. The mathematical equation for PPV is given in equation 12 in Figure 3. Similarly, the NPV is the probability that the negatively identified case is negative. The mathematical equation is given in equation 13 in Figure 3.

#### AUROC

AUROC is a performance measure by which we can evaluate the performance of the model. AUROC is a performance matrix for *discrimination*; it shows the predictive model’s ability to discriminate between positive and negative cases.

## Results

### Dataset Characteristics

A total of 9214 prescriptions with an alert were collected during the 10-month study period. After preprocessing and removing duplicate prescriptions with the same registration numbers, 6453 prescriptions were used to develop our models. The neurology department got the highest number of alerts (1039/6453, 16.10%). Of those alerts, 546 (52.55%) were accepted and 493 (47.45%) were rejected by physicians (see Multimedia Appendix 1, Figure S1). The urology, dermatology, chest medicine, family medicine, metabolism, and otolaryngology departments observed higher alert rates of 10.61% (685/6453), 9.80% (633/6453), 6.91% (446/6453), 6.61% (427/6453), 6.52% (421/6453), and 6.50% (420/6453), respectively. Moreover, eight departments, including rehabilitation medicine, infectious disease, and ophthalmology, had alert rates of more than 1%. Gender, diagnosis codes, disease codes, physician IDs, and department IDs were used to develop and validate our prediction model (see Table 1).

### Prediction Performance of Machine Learning Algorithms

**Figure 4:**
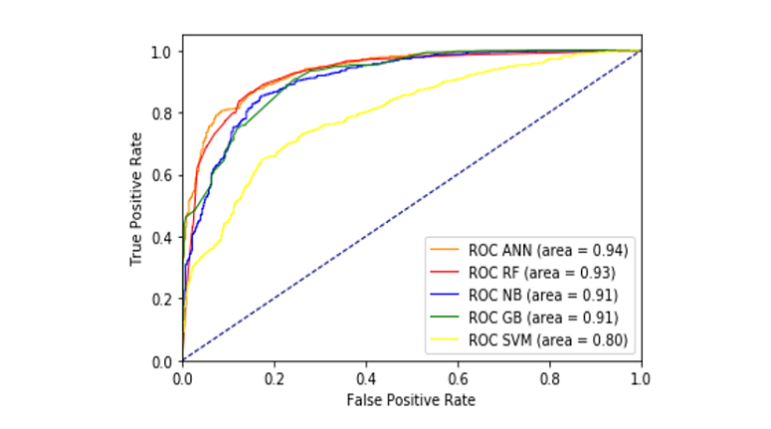
Performance of machine learning prediction models. ANN: artificial neural network; GB: gradient boosting; RF: random forest; NB: naïve Bayes; ROC: receiver operating characteristic; SVM: support vector machine.

We developed five types of machine learning models to predict physician response. To determine the overall performance of predictive models, six evaluation metrics were applied. Among all the machine learning models, ANN showed the best performance (AUROC 0.94) (see Figure 4 and Multimedia Appendix 1, Figure S2).

The accuracy of the ANN, RF, NB, GB, and SVM models were 0.88, 0.85, 0.83, 0.82, and 0.57. The sensitivity and specificity of the ANN, RF, NB, GB, and SVM models were 0.87, 0.88, 0.87, 0.79, and 0.57 and 0.83, 0.82, 0.78, 0.90, and 1.0, respectively (see Table 2).

**Table 2:** Performance of the prediction models.

Algorithm	Accuracy	Sensitivity, %	Specificity, %	PPV^a^, %	NPV^b^, %	F1
Artificial neural network	0.885	87.01	83.46	87.84	82.40	87.42
Random forest	0.857	88.29	82.48	86.62	84.57	87.44
Naïve Bayes	0.835	87.48	78.74	83.22	84.03	85.29
Gradient boosting	0.828	79.46	90.24	94.59	67.15	86.36
Support vector machine	0.575	57.45	100.0	100.0	0.54	72.97

^a^PPV: positive predictive value.

^b^NPV: negative predictive value.

## Discussion

### Principal Findings

CDSSs directly assist physicians in making correct clinical decisions that ultimately reduce prescription errors by generating real-time alerts and lessen probable unwanted consequences. Clinical workflow is often impaired by excessive numbers of alerts; therefore, physicians pay less attention to alerts and even ignore alerts indiscriminately. This study focused on physicians’ recent practice patterns and represented the findings of machine learning models to predict physicians’ responses to alerts from a disease medication–related CDSS. The key findings are as follows: (1) an ANN model can correctly predict physicians’ responses with higher accuracy than other models and (2) we identified potential features that could provide insight into the system design. These findings may contribute to building a sophisticated provider-friendly interface in which a CDSS may offer real-time alerts if the prediction is positive for that individual physician. If the prediction is negative, that means physicians might not accept the alert; therefore, the CDSS will not generate alerts during the prescribing of prescriptions or will provide soft or passive alerts without interruption. However, all the alerts would be recorded and the report sent to the individual physician by email on a weekly basis to inform them of how important the alerts were in order to reduce unwanted consequences.

### Clinical Implications

CDSSs have already shown their capability to improve patient safety and quality of care by lowering the number preventable medication errors [32-34]; however, an unreasonable override rate raises questions regarding the quality of CDSSs. Patient safety and effective care could be improved by initiating sophisticated criteria for generating alerts in the CDSS that prevent alert fatigue and minimize the override rate [35-37]. Identification of physicians and departments who override alerts more often would help to reduce the override rate and help us understand how physicians would respond to drug-disease alerts, which would result in immense benefits. There are no previous studies that used a machine learning prediction model to identify physicians and departments who override alerts more often. In this study, machine learning algorithms were used to reduce alert fatigue by identifying physicians and departments who override alerts more often. Our findings are consistent with existing research that showed physicians played a great role in alert override [38]. Bell et al showed that alert override can be minimized by physicians’ preferences for alert selection [39]. There are several reasons that can make physicians override alerts. First, current medication-related CDSSs are not designed to take the patient’s previous medication history into account. Sometimes patients are already tolerant of the drug and physicians need to override the alert and prescribe the drug [40]. Second, some CDSSs required an entry for the reason for alert override and that lead to an unacceptable time burden for physicians [41]. Third, physicians believe that *they already know* the alert is inappropriate based on their experience, so they are more likely to override the alert [13,42]. Our study also provides a very important point: no matter how accurate the CDSS is according to the most relevant knowledge base, the alert acceptance was highly affected by the individual physician’s perspective. Our model will reduce the gap between real-world clinical practice and knowledge-based theory.

Yeh et al [43] demonstrated that dermatology, gynecology-obstetrics, family medicine, and ophthalmology departments had higher acceptance rates; however, pediatrics, psychiatry, and internal medicine departments, such as cardiology, endocrinology and metabolism, gastroenterology, hematology, rheumatology, and general medicine, had lower acceptance rates. In our study, we also found that physicians’ decisions vary from department to department.

### Strengths and Limitations

This study has several strengths. First, this is the first study to use machine learning algorithms to predict physicians’ intentions to accept or reject alerts. This model may help to reduce alert fatigue in the current CDSS. Second, this study is personalized for each physician. Third, the performance of the model is satisfactory, such that it would help to reduce alert fatigue. Despite several strengths, our study also has several limitations that need to be addressed. First, we did not include free-text override reasons in our analysis, and free-text reasons could add additional value to our model. However, our model provided the AUROC with decent specificity and sensitivity. Second, we did not include physicians' experiences, working periods, ages, and genders in this prediction model. These data are difficult to collect retrospectively because EHR systems do not record this type of information. Third, we have only used one hospital dataset; multiple hospital datasets would make our model more reliable.

### Future Works

**Figure 5:**
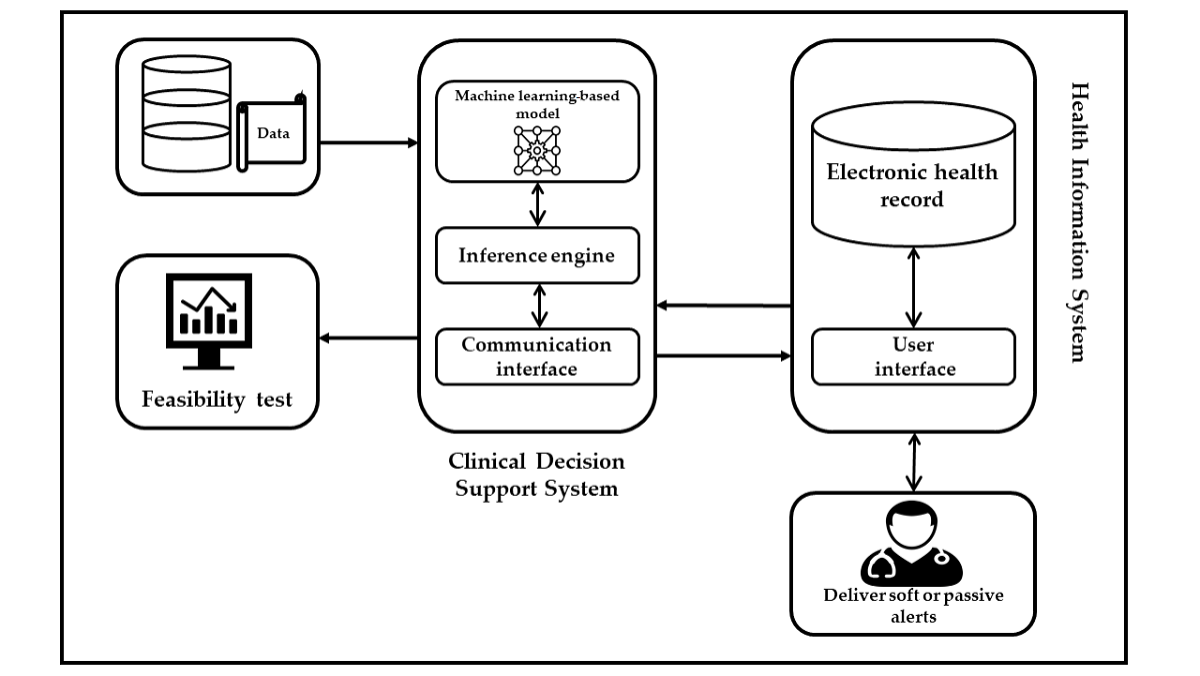
Future direction of this study.

This was the first part of our work. In the future, we will integrate our prediction model into the CDSS in order to check the feasibility of our model. It will help to reduce alert fatigue and result in a sophisticated CDSS by providing soft or passive alerts. Moreover, we will also try to get feedback from physicians about our prediction model (see Figure 5).

### Conclusions

The findings of the study showed the potential for machine learning prediction models to predict physicians’ responses with high sensitivity and specificity. Among the five machine learning algorithms, the ANN model showed greater performance than the other models. This model can be a promising tool to reduce alert fatigue from CDSSs in clinical settings and can help to correctly identify an individual’s alert acceptance rate.

## Appendix

Multimedia Appendix 1 Description of alerts in different departments and problem fitting checks.

## Abbreviations

AESOP: AI-Enhanced ​Safety of ​Prescription
ANN: artificial neural network
ATC: Anatomical Therapeutic Chemical
AUROC: area under the receiver operating characteristic curve
CDSS: clinical decision support system
CPOE: computerized physician order entry
EHR: electronic health record
GB: gradient boosting
ICD-10-CM: International Classification of Diseases, 10th Revision, Clinical Modification
MOE: Ministry of Education
MOST: Ministry of Science and Technology
NB: naïve Bayes
NPV: negative predictive value
PPV: positive predictive value
ReLU: rectified linear unit
RF: random forest
SVM: support vector machine
