# Supplementary material for: Machine Learning Approach to Reduce Alert Fatigue Using a Disease Medication–Related Clinical Decision Support System: Model Development and Validation
Source: JMIR Med Inform. 2020 Nov 19;8(11):e19489. doi: 10.2196/19489 (PMC7714650; doi:10.2196/19489)

**Supplementary figures:**

**Figure S1**: Distribution of alerts in different department

**Figure S2:** Outfitting and under fitting problem checking


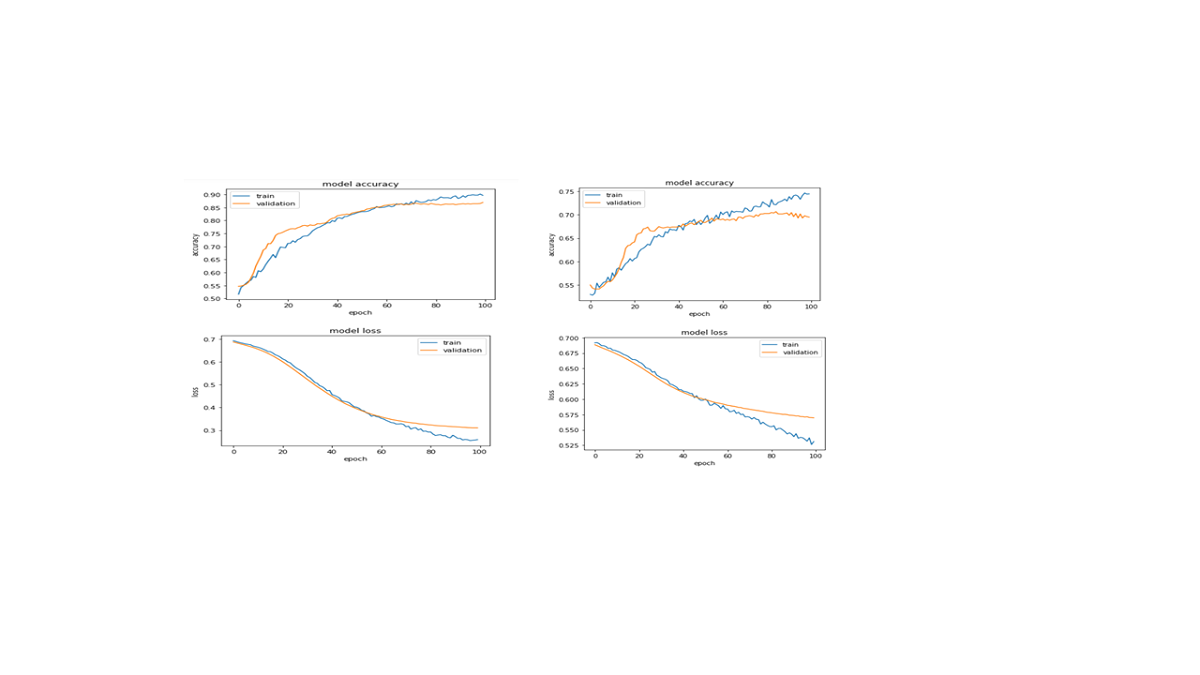

Supplement: Multimedia Appendix 1 [file medinform_v8i11e19489_app1.docx]
